# Telemetric left ventricular monitoring using wireless telemetry in the rabbit model

**DOI:** 10.1186/1756-0500-4-320

**Published:** 2011-09-05

**Authors:** Mallory K Tate, William S Lawrence, Randy L Gourley, Diana L Zavala, Lori E Weaver, Scott T Moen, Johnny W Peterson

**Affiliations:** 1 Galveston National Laboratory, University of Texas Medical Branch, Galveston, Texas, USA; 2 Animal Resources Center, University of Texas Medical Branch, Galveston, Texas, USA; 3 Department of Microbiology and Immunology, University of Texas Medical Branch, Galveston, Texas, USA

## Abstract

**Background:**

Heart failure is a critical condition that affects many people and often results from left ventricular dysfunction. Numerous studies investigating this condition have been performed using various model systems. To do so, investigators must be able to accurately measure myocardial performance in order to determine the degree of left ventricular function. In this model development study, we employ a wireless telemetry system purchased from Data Sciences International to continuously assess left ventricular function in the rabbit model.

**Findings:**

We surgically implanted pressure-sensitive catheters fitted to wireless radio-transmitters into the left ventricle of Dutch-belted rabbits. Following recovery of the animals, we continuously recorded indices of cardiac contractility and ventricular relaxation at baseline for a given time period. The telemetry system allowed us to continuously record baseline left ventricular parameters for the entire recording period. During this time, the animals were unrestrained and fully conscious. The values we recorded are similar to those obtained using other reported methods.

**Conclusions:**

The wireless telemetry system can continuously measure left ventricular pressure, cardiac contractility, and cardiac relaxation in the rabbit model. These results, which were obtained just as baseline levels, substantiate the need for further validation in this model system of left ventricular assessment.

## Background

In the case of left ventricular (LV) dysfunction, the myocardium surrounding the left ventricle is functionally disrupted, thereby causing impaired contraction and/or relaxation. The result is ineffective pumping and inadequate blood flow. Impaired left ventricular systolic function and/or impaired left ventricular diastolic function leads to congestive heart failure (CHF), which is a condition that is increasing in prevalence worldwide [1-3]. Consequently, LV dysfunction is a topic under intense investigation by the scientific community.

The degrees of contraction and relaxation are both used to assess overall LV function. With regards to the contractile phase, many have used cardiac contractility as a factor to gauge LV systolic function [4-7]. Myocardial contractility is the intrinsic ability of cardiac muscle to develop force at a given muscle length, and it increases upon sympathetic stimulation. Frequently, cardiac contractility is expressed as the maximum change in pressure divided by the change in time (dP/dt_max_) [6,8-11], and it denotes the maximum rate of increase in pressure during isovolumic contraction. Another parameter reported as a useful index of contractility is V_max _[12-14]. This is the maximal velocity of contractile shortening, and it has the added advantage of being load-independent. Ventricular relaxation, on the other hand, is used to assess LV diastolic function. It is often times expressed either as the maximum (negative) change in pressure divided by the change in time (-dP/dt_max_) [15-19], which represents the decrease in pressure during isovolumic relaxation, or as Tau [16,18,20,21], the time constant of LV relaxation. Since determining ventricular contractility and relaxation requires one to know ventricular pressure during full cardiac cycles, investigators, using various animal models, utilize pressure-sensitive catheters which are implanted into the animals' ventricle [22-26]. In an ideal model system, the catheterization is both well-tolerated by the animal as well as accurate in pressure reading. Moreover, an ideal system allows for the animal to remain in an unrestrained, conscious state during monitoring.

In this model development study, we test the utilization of wireless telemetry for determining various LV parameters, including left ventricular pressure (LVP), cardiac contractility, and the degree of ventricular relaxation, in the rabbit model. This wireless system is beneficial in that the animal is not restrained with a wire harness and is fully conscious which more accurately mimics normal cardiovascular conditions. The rabbit model has been used extensively in cardiovascular research, however, to our knowledge there are no reports describing the use of wireless telemetry to monitor LV function in specifically this animal model. Here we present baseline data that demonstrates the usefulness and feasibility of wireless, telemetric monitoring for determining the degree of LV function in conscious, unrestrained Dutch-belted rabbits. Consequently, this model system warrants further validation using physiological challenges and/or pharmacological interventions.

## Methods

### Rabbits and housing

Specific-pathogen-free female Dutch-belted dwarf rabbits, 7 to 8 weeks in age and weighing 1.5 to 2.0 kg, were purchased from Myrtle's Rabbitry (Thompson Station, TN). The vendor's comprehensive health assessment, which includes serologic testing, indicated that the animals were free from *Pasteurella multocida*, *Pasteurella pneumotropica*, *Bordetella bronchiseptica*, *Treponema cuniculi*, *Clostridium piliformis*, cilia-associated respiratory bacillus, oral papillomavirus, arthropod ectoparasites, helminth endoparasites, and protozoans. Upon delivery, the animals were pair- or singly housed at the University of Texas Medical Branch Animal Resources Center in stainless steel, ventilated rabbit racks (Allentown, Allentown, NJ) and allowed to acclimate for one week. The animal room was maintained on a 12:12-hr light:dark cycle, with the temperature range at 19 to 22°C and the humidity between 30% and 70%. The rabbits were fed approximately 170 g commercial chow (Rabbit Diet 5321, LabDiet, Richmond, IN) daily and given water *ad libitum*. All animal procedures were conducted under protocols approved by the University of Texas Medical Branch Institutional Animal Care and Use Committee.

### Telemetry system and surgical implantation

The telemetry equipment, purchased from Data Sciences International (DSI) (St. Paul, MN), included an implantable transmitter (model TL11M3-D70-PCTP), a receiver (model RMC1), a data processing device (Data Exchange Matrix), and an ambient pressure reference monitor (APR-1). The transmitter has two pressure catheters and two biopotential leads, however, only one pressure catheter was used. The unused catheter/leads were immobilized near the body of the transmitter. The pressure catheter is a 14-cm fluid filled catheter with a terminal sensing region containing a non-compressible fluid and a plug of biocompatible gel. This sensing region relays pressure waves to the DSI transmitter. Calibration of the transmitter is accomplished by placing it into a sealed pressure chamber with measurements taken at 750, 850, and 950 mmHg. The output is read as a raw frequency output and is used as the calibration values for the transmitter. The data for each rabbit were recorded approximately two weeks after surgery, with the exact numbers of days being dependent on the animals' level of recovery. The data acquisition system, which has a sampling frequency of 500 Hz, was programmed to record data continuously for 72 h, with the data recorded every 20 s. This was later used to compute 1-h moving averages. The parameters that were continuously monitored were heart rate, mean left ventricular pressure (LVP), left ventricular systolic pressure (LVSP), left ventricular end diastolic pressure (LVEDP), dP/dt_max_, V_max_, -dP/dt_max_, and Tau. LVSP is defined as the peak pressure after the detected dP/dt_max_, while LVEDP is defined as the pressure at the end of diastole. Also, Tau is defined as the relaxation time constant from -dP/dt_max _to the point where the pressure has dropped 66% of the distance from systolic to diastolic pressure. All data was computed using an analysis program (Dataquest 4.1, Data Sciences International, St. Paul, MN), and all graphs were prepared in Excel (Microsoft).

The rabbits were given ketamine-HCl (50 mg/kg IM) (Fort Dodge Animal Health, Fort Dodge, IA), buprenorphine (0.05 mg/kg IM) (Hospira, Inc, Lake Forest, IL), and glycopyrrolate (0.1 mg/kg IM) (American Regent, Inc., Shirley, NY) as pre-anesthetics prior to intubation. They were intubated with a 2.5 mm cuffed endotracheal tube using the blind technique, and they were inducted under 3% isoflurane (Webster Veterinary, Sterling, MA) and maintained at 2-2.5% during the surgical procedure. A catheter was placed in the left ear vein for fluid replacement with Lactated Ringers solution (Baxter, Deerfield, IL) at a rate of 30 ml/kg/hr. The temperature, ECG, blood pressure and pulse oximeter probes were placed on the animals and measured using the Surgivet (Smith Medical, Inc., Waukesha, WI). The rabbits were supplemented with heat using a heating pad (Gaymar Industries, Inc., Orchard Park, NY). Once the thoracic cavity was opened, the rabbit was ventilated at 15 to 30 breaths per minute until the cavity was closed.

**Figure 1:**
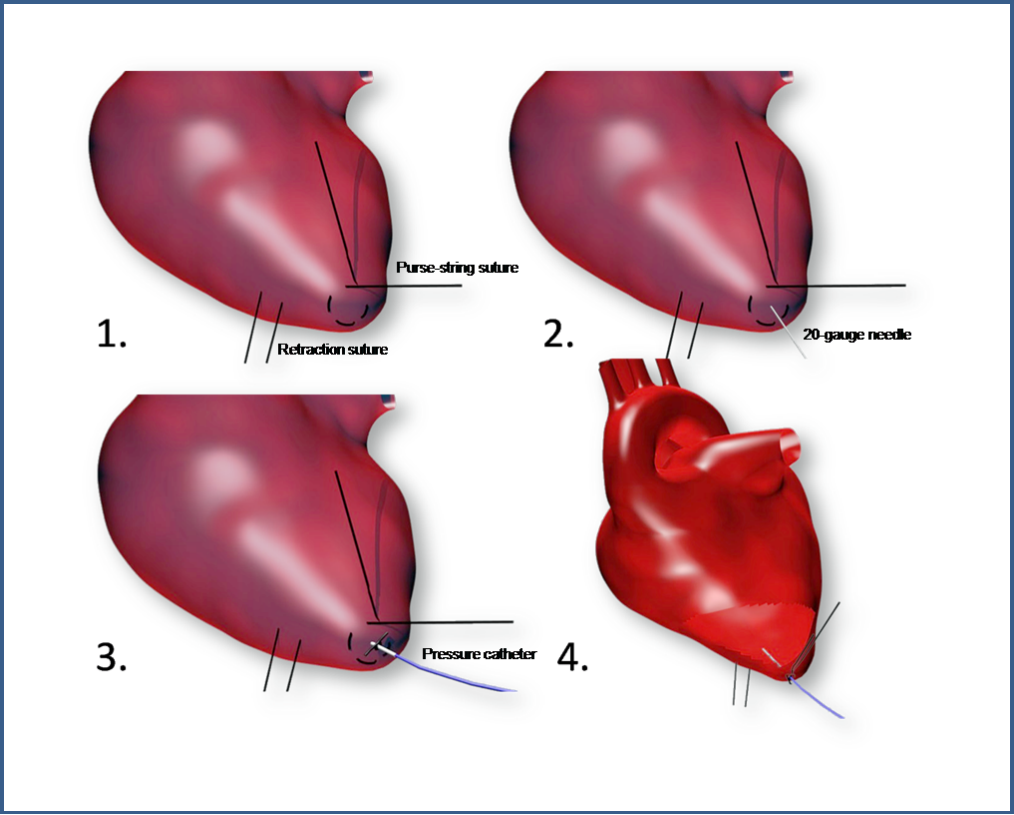
**Surgical procedure**. Placement of pressure catheter into the left ventricular cavity.

The left side of the animals was shaved, and the surgical site was disinfected with betadine and alcohol three times. The muscle layer over the 5^th ^intercostal space was injected with 1% buprivicaine (approximately 0.5 ml) (Hospira, Inc., Lake Forest, IL), with each injection spaced 5 cm apart from the cervical to thoracic region. An incision for the transmitter body was placed on the left lateral side of the abdominal wall approximately 4 cm behind the last rib. The telemetry transmitter was placed in a subcutaneous pocket behind the last rib over the abdominal region, after which it was secured with an anchor suture. A skin incision was then made over the 5^th ^intercostal space, and the pressure catheter of the transmitter was tunneled subcutaneously to the incision. The incision over the transmitter was closed with 3-0 Vicryl in a simple continuous pattern. Another 3-0 Vicryl suture was gently placed around the end of the pressure catheter. Following, a thoracotomy was performed at the 5^th ^intercostals space on the left side of the animal. With the ribs retracted and the apex of the heart accessible, the pericardium was incised, and a retraction suture (6-0 prolene), which was removed after catheterization, was placed superficially through the myocardium just left of the intended catheterization point (Figure 1). Using the retraction suture to stabilize the heart, a purse-string suture was placed around the apex of the left ventricle which was then perforated using a 20-gauge needle (Figure 1). The pressure catheter was inserted into the left ventricle and advanced until the anchor suture on the catheter met the myocardium. The purse-sting suture was then tightened at the apex, and the catheter was fixed using Vetbond (Veterinary Products Laboratories, Phoenix, AZ). Lastly, the lungs were manually ventilated and checked for functionality, and the animals' ribs, muscle layer, and skin layer were closed in routine fashion.

The rabbits, which were singly housed following surgical implantation, were checked for any abnormal breathing, pain, or distress. Following the surgery, heat was provided until the rabbits regained normal body temperature. Buprenorphine was given for the first 48 hours, and the animals were monitored very closely until they were eating and drinking normally (approximately 2 weeks).

## Results

### Heart rate and Left ventricular pressures

**Figure 2:**
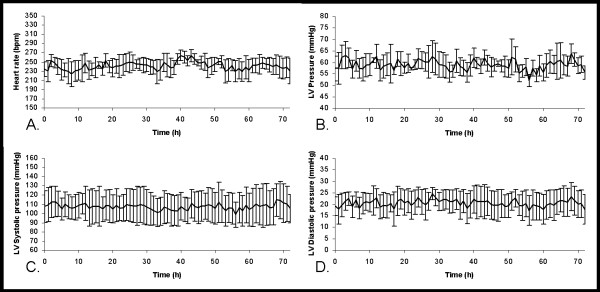
**Heart rate and left ventricular pressures**. A 72-hr continuous trace of baseline heart rate and left ventricular pressures in rabbits with telemetry monitoring devices. Data presented as a 1-hr moving average with bars indicating standard error (**A**) heart rate. (**B**) left ventricular pressure. (**C**) left ventricular systolic pressure. (**D**) left ventricular end diastolic pressure.

**Figure 3:**
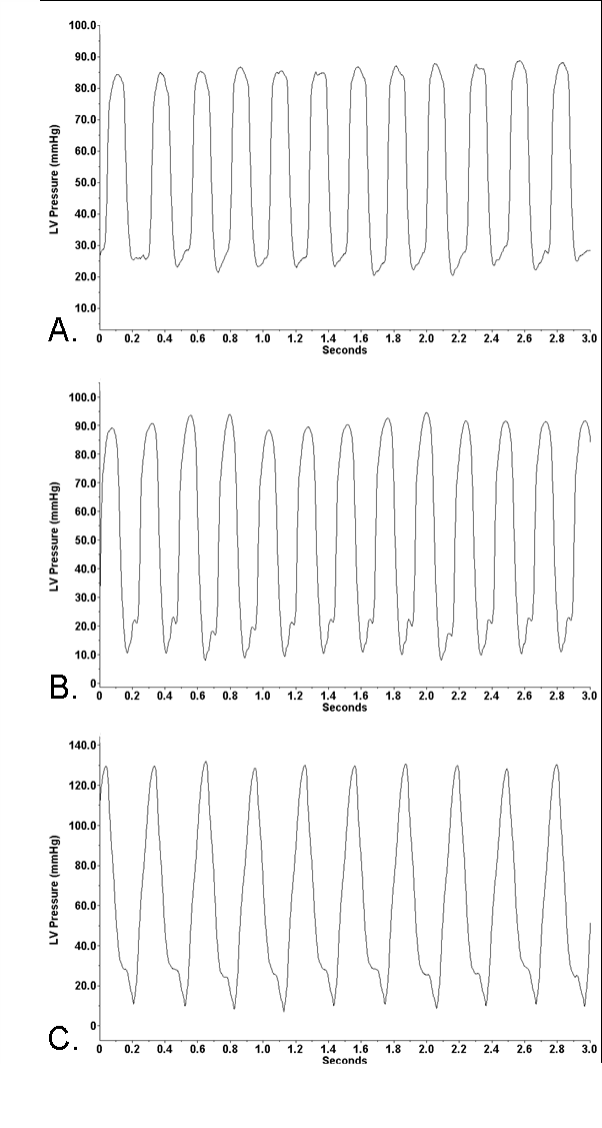
**Left ventricular pressure waveforms**. The left ventricular pressure waveforms of rabbits during telemetric monitoring. (**A**) rabbit 1. (**B**) rabbit 2. (**C**) rabbit 3.

The 72-hr heart rate and LVP data are presented as 1-hr moving averages. We performed surgery on four rabbits, however, the data from one rabbit was excluded due to the presence of a cardiac arrhythmia thought to have been caused by the surgery. The remaining three animals displayed normal heart rates (200-300 bpm [27]) throughout the recording period, with the average ranging from 229 to 264 bpm (Figure 2A). The rabbits' average LVP varied from 52 to 63 mmHg (Figure 2B), with the average LVSP (Figure 2C) and LVEDP (Figure 2D) ranging from 99 to 114 mmHg and 17 to 24 mmHg, respectively. The waveform illustrations, which were randomly selected from the recorded 72-hr waveform data during data analysis, show the LVP waveforms of the three rabbits over a 3-sec time interval (Figure 3A-C).

### Indices of contractility and relaxation

**Figure 4:**
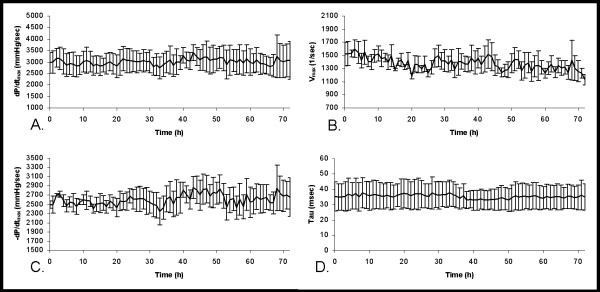
**Left ventricular contractility and relaxation indices**. A 72-hr continuous trace of baseline contractility and relaxation indices in rabbits with telemetry monitoring devices. Data presented as a 1-hr moving average with bars indicating standard error (**A**) dP/dt_max_. (**B**) V_max_. (**C**) -dP/dt_max_. (**D**) Tau.

All contractility and relaxation data are presented as 1-hr moving averages. The average dP/dt_max _for the three rabbits remained fairly constant (Figure 4A), however, rabbit 2 had a dP/dt_max _that was notably higher than the other two rabbits on each day (Table 1). The V_max _for the three animals was comparable each day (Table 1), but when averaging the three together, there appeared to be some variability from one hour to the next at various times (Figure 4B). The average -dP/dt_max _for the three rabbits also remained fairly constant throughout the recording period, with the variability increasing at the later time points (Figure 4C). Lastly, the Tau varied moderately between the three animals (Figure 4D), possibly due to the higher values recorded in rabbit 3 (Table 1).

**Table 1:** Contractility and relaxation indices of rabbits on each day for 3 days

		Rabbit 1			Rabbit 2			Rabbit 3	
	**Day 1**	**Day 2**	**Day 3**	**Day 1**	**Day 2**	**Day 3**	**Day 1**	**Day 2**	**Day 3**

dP/dtmax (mmHg/sec)	3081 ± 42	2908 ± 50	2735 ± 27	3702 ± 33	3879 ± 51	4126 ± 74	2116 ± 23	2342 ± 42	2228 ± 34

Vmax (1/sec)	1338 ± 19	1138 ± 10	1098 ± 9	1615 ± 43	1534 ± 38	1535 ± 36	1339 ± 20	1489 ± 29	1309 ± 30

-dP/dtmax (mmHg/sec)	2385 ± 32	2122 ± 34	2099 ± 17	2785 ± 23	2978 ± 30	3067 ± 52	2464 ± 35	2811 ± 45	2742 ± 48

Tau (msec)	29 ± 0.4	31 ± 0.2	31 ± 0.2	24 ± 0.3	23 ± 0.2	22 ± 0.1	54 ± 0.4	49 ± 0.9	50 ± 0.4

Data presented as average ± standard error

## Discussion

Telemetry has been used extensively in various animal models in the past with the purpose of investigating cardiovascular disorders. The use of telemetry in the murine model was recently reported by investigators assessing right ventricular systolic pressure and heart rate in mice treated with an experimental therapeutic aimed at reducing pulmonary hypertension [28]. In this murine study, the investigators introduced a pressure catheter into the right ventricle of the mice by way of the right jugular vein. Likewise, telemetry has been used for determining cardiac function, specifically contractility, in rats [22]. In this case, the investigators were able to determine the rats' QA interval, an indirect indicator of contractility, by surgically implanting ECG and pressure leads. In addition to the rodent models, animal models involving higher organisms have also been used in conjunction with telemetry, namely dogs, pigs, and non-human primates [23,25,26]. Nonetheless, we report here using dwarf Dutch-belted rabbits which have the added advantages of being more similar to humans relative to rodents, and being more cost-effective and easy to handle relative to non-human primates and other higher organisms. There are past studies which involved surgically placing a pressure transducer in the heart of rabbits in order to evaluate LV function, however, in these cases, the systems allowed only limited movement since the transducers used were physically connected to an acquisition/storage system [24,29]. Our model system allows the rabbits to remain unrestrained and fully conscious.

The values we recorded for the various parameters are similar to those reported in previous studies that used either an alternate method, such as echocardiography, or different non-wireless hardware, for assessing LV function in naïve rabbits [24,29-31]. Any differences between our values and the values of these previous studies could be attributed to the use of a different rabbit strain, which often times was the New Zealand White strain. Some discrepancies could also be due to the fact that in some of these previous studies the animals were under anesthesia while being monitored. Additionally, the surgical procedure we present here, while beneficial for directly measuring LV pressures, could bring about slightly altered values for LV function, which is probable with an invasive procedure of this type. Also, proper placement of the pressure catheter tip, which is directly at the apex, is crucial for assuring that the tip is directly in the ventricular chamber. Any contact of the catheter tip with the muscle wall (can occur due to animal movement), or any blood-flow obstruction, could lead to abnormal pressure waveforms which in turn would result in atypical pressure values. This could have occurred with rabbit 3, which would possibly explain the lower dP/dt_max _relative to the remaining two animals and the slightly different waveform. Then again, both the -dP/dt_max _and V_max _from rabbit 3 was closely similar to rabbits 1&2, suggesting that the pressure waveform of rabbit 3 used to calculate the parameters was normal. Also worth noting is that our dP/dt_max _(and -dP/dt_max_) are derived from pressure measurements which makes it load-dependent. Fortunately, this system is able to denote contractility as V_max _as well, which is reported to be a load-independent variable [14]. Future studies that involve altering cardiac physiology (heart rate, preload, afterload, and etc.) by means of drug administration and/or surgical manipulation would be beneficial for testing this model system further.

## Conclusions

The DSI telemetry system allowed us to record various parameters (at baseline) that are indicative of the degree of LV function. The values we present here were recorded in Dutch-belted rabbits that were both fully conscious and unrestrained. Monitoring the animals in this state lessens the role that either stress due to restraint or anesthesia might play in attaining accurate results. Overall, this report supports the use of wireless telemetry in assessing LV function in the rabbit model.

## List of abbreviations

CHF: Congestive heart failure; DSI: Data Sciences International; LV: Left ventricular; LVP: Left ventricular pressure; LVSP: Left ventricular systolic pressure; LVEDP: Left ventricular end diastolic pressure.

## Competing interests

The authors declare that they have no competing interests.

## Authors' contributions

MT and WL initiated the study, performed the surgeries, and analyzed the data. RG, DZ, and LW assisted in the surgeries and were responsible for animal care. SM collected/analyzed the data. JP supervised the study. All authors took part in preparation of the manuscript. All authors have read and approved the final manuscript.
